# Advances in nanotechnology for the treatment of GBM

**DOI:** 10.3389/fnins.2023.1180943

**Published:** 2023-05-05

**Authors:** Dongyan Wei, Ni Zhang, Shuang Qu, Hao Wang, Jin Li

**Affiliations:** ^1^Department of Psychiatry, West China Hospital, Sichuan University, Chengdu, China; ^2^College of Life Sciences, Tarim University, Alar, China; ^3^Mental Health Center, West China Hospital, Sichuan University, Chengdu, China; ^4^College of Life Science and Technology, Beijing University of Chemical Technology, Beijing, China

**Keywords:** malignant glioma, nanotechnology, tumor diagnosis, tumor therapy, targeted therapy

## Abstract

Glioblastoma (GBM), a highly malignant glioma of the central nervous system, is the most dread and common brain tumor with a high rate of therapeutic resistance and recurrence. Currently, the clinical treatment methods are surgery, radiotherapy, and chemotherapy. However, owning to the highly invasive nature of GBM, it is difficult to completely resect them due to the unclear boundary between the edges of GBM and normal brain tissue. Traditional radiotherapy and the combination of alkylating agents and radiotherapy have significant side effects, therapeutic drugs are difficult to penetrate the blood brain barrier. Patients receiving treatment have a high postoperative recurrence rate and a median survival of less than 2 years, Less than 5% of patients live longer than 5 years. Therefore, it is urgent to achieve precise treatment through the blood brain barrier and reduce toxic and side effects. Nanotechnology exhibit great potential in this area. This article summarizes the current treatment methods and shortcomings of GBM, and summarizes the research progress in the diagnosis and treatment of GBM using nanotechnology.

## 1. Introduction

The World Health Organization (WHO) divides glioma into low grade and high grade, according to the malignancy degree of tumor (Chen R. et al., 2017). High grade gliomas, also known as malignant gliomas, are highly invasive and difficult to cure and are therefore associated with high mortality. Glioblastoma multiforme (GBM) is the most malignant glioma in the brain (Gao and Jin, 2014; Saenz del Burgo et al., 2014; Bredlau et al., 2017a). The standard first-line treatment regimen for GBM includes maximum surgical resection and postoperative radiotherapy with temozolomide (Gallego and Ceña, 2020; Śledzińska et al., 2021). Because of the highly invasive nature of GBM, surgeons often cannot determine whether a tumor was completely removed, resulting in a high postoperative recurrence rate (Stylli et al., 2015; Xie et al., 2015; Faustino et al., 2020); The hypothesis of cancer stem cells (CSCs) suggests that stem cell-like properties make CSCs resistant to cytotoxic therapy, resulting in overall drug resistance, and significantly increasing the recurrence rate after surgery. Another complicating factor is the existence of the blood brain barrier (BBB). The protection afforded by anatomical feature means that only a small portion of chemotherapy drugs that enter the body through radiotherapy enters the brain, so the treatment process is potentially more toxic to healthy tissues than to the tumor itself (Zhang et al., 2015).

**Figure 1:**
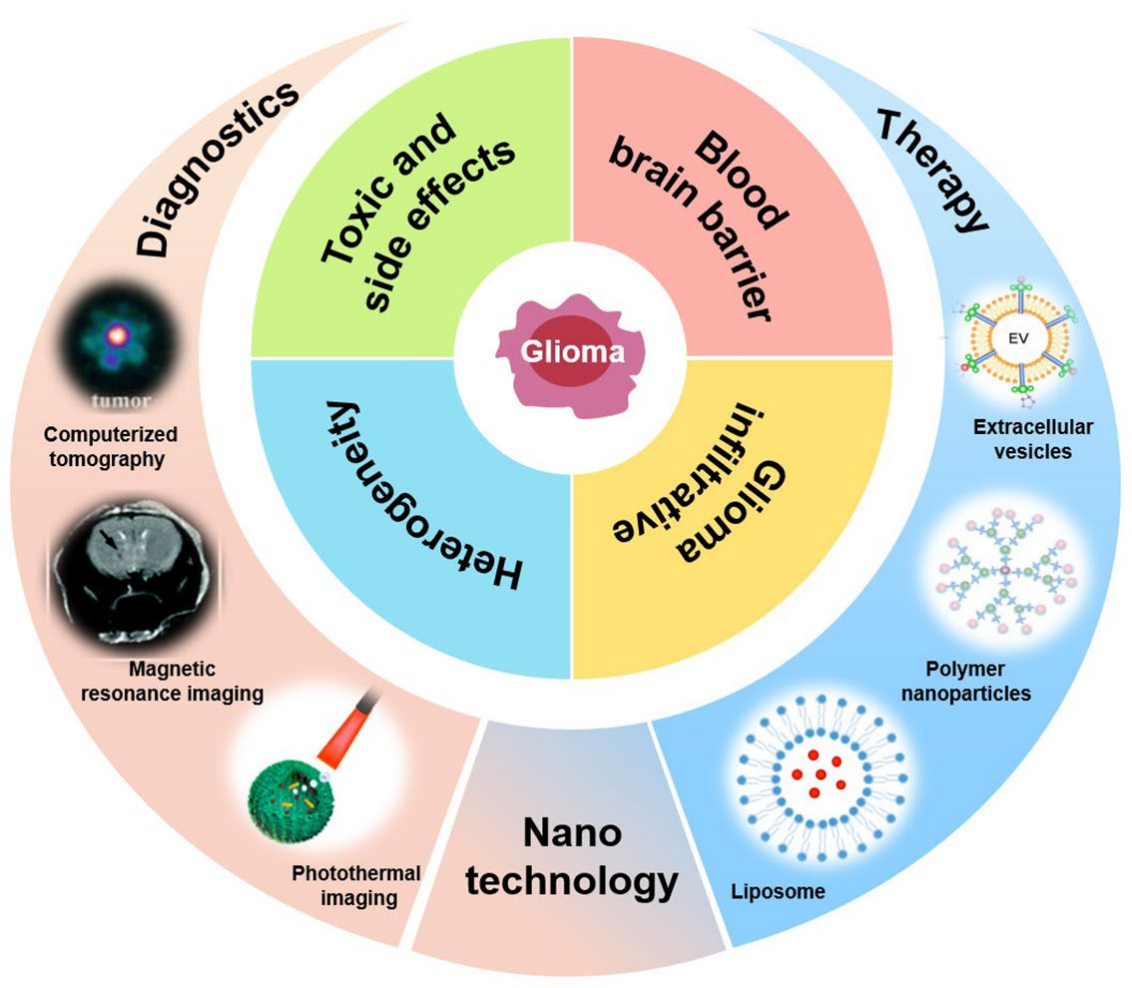
Nanodiagnosis and nanotherapy of GBM.

With the rapid development of nanotechnology, a growing number of studies have used nanotechnology to provide new options for the diagnosis and treatment of GBM (Kim et al., 2015). Nanoparticle delivery systems offer a wealth of strategies that solve the problem presented by the BBB and can achieve targeted therapies. In addition, research regarding nano-imaging technology for diagnosis, and using diagnostic and therapeutic nanoparticles to achieve precise treatment of GBM is in full swing (Bredlau et al., 2017b; Figure 1).

In this review, we will introduce and discuss the research progress related to nanomaterials as developed for precise diagnosis and precise treatment of GBM.

## 2. The role of nanomaterials in the treatment of GBM

With the continuous development of nanotechnology, researchers continue to research and develop new nanoscale diagnosis and treatment strategies for GBM. In the diagnosis and imaging of GBM, compared to conventional enhancer imaging, nanoparticles can enhance contrast sensitivity, binding affinity, and targeting specificity. When combined with nanoparticles, drugs can cross the blood brain barrier, enabling the detection of lesions that are difficult to detect, and achieving precise diagnosis. During the treatment of GBM, the nanocarrier platform can not only transport small molecule drugs, but also transport macromolecular drugs such as proteins, polypeptides, and genes to actively or passively target GBM cells, achieving better tissue penetration through the blood brain barrier, better drug release, lower toxicity, and more precise treatment. Nanoparticles are made into drug carriers such as shells or cages to encapsulate chemotherapy drugs and specifically present them to GBM, greatly improving the therapeutic effect. For example, paclitaxel (PTX) has a good inhibitory effect on GBM *in vitro*, but it will be restricted by the blood brain barrier during systemic administration. PTX-PLGA nanoparticles coated with polyethylene glycol (PEG) can well stay in the blood circulation and increase their bioavailability.

## 3. Precise diagnosis of GBM with nanotechnology

Precise diagnosis is an essential component to successful cancer treatment, and diagnosis is particularly challenging in the context of GBM due to its location deep in brain tissue. To achieve high sensitivity, high specificity, high resolution and deep tissue penetration, researchers are actively developing a variety of imaging techniques and contrast agents (Tang et al., 2019). The small particle sizes of nanomaterials, which include natural or synthetic particles with diameters between 1 and 1,000 nm (Manek and Petroianu, 2021), endow them with an important advantage. In addition, nanoparticles can have properties that differ from those of traditional drugs, such as photosensitivity and magnetism. Currently, a variety of nanoparticles have been developed to label glioblastoma through the blood brain barrier, and then accurately image it. At the same time, they can carry a large amount of radioactive isotopes, improving the specificity and sensitivity of imaging, and achieving the visualization of glioblastoma. Multimodal imaging using nanotechnology for percise diagnosis can contribute greatly to the success of surgery and the accuracy of targeted therapy (Bredlau et al., 2017b).

The most commonly used medical imaging methods for GBM are magnetic resonance imaging (MRI), computer tomography (CT), and optical imaging. In order to improve the accuracy and sensitivity of imaging, contrast agents are usually needed. Nanomaterials are well suited for this role because they tend to have high loading capacities and low toxicity. They are also amenable to simple surface modifications that allow the preparation of multifunctional materials to create long-lasting and low toxicity contrast agents for multimodal imaging or simultaneous treatment and diagnosis (Lee et al., 2013). Accordingly, there has been a recent surge in research attempting to integrate diagnosis and treatment by using the properties of nanomaterials as both drug carriers and contrast agents.

### 3.1. Super paramagnetic iron oxide: a nanoprobe for super magnetic resonance imaging

Magnetic resonance imaging (MRI) allows the imaging of biological tissues by measuring the interactions of radio frequency pulses with magnetic fields to generate images of internal organs (Jin et al., 2020). Its high spatial resolution in the absence of radiation make it particularly effective at soft tissue imaging, and it has become the main tool for diagnosis and postoperative observation of GBM. The obvious disadvantages of MRI are low sensitivity and poor ability to distinguish between diseased and healthy tissue, and matching contrast agents are often used to improve the contrast effect (Jeon et al., 2021). The current mainstream MRI contrast agents are magnetic nanomaterials (Liang et al., 2018). Magnetic nanoparticles have ultra-high magnetic mobility, good biological compatibility, and the easy modification of targeted carriers has led to their decoration with effective contrast agents (Lu et al., 2007; Etoc et al., 2015; Storey et al., 2020). Superparamagnetic iron oxide nanoparticles (SPION) (Bulte and Kraitchman, 2004; Stoll and Bendszus, 2009; Schweiger et al., 2010), commonly used as magnetic nanoparticles for biomedical applications, are another MRI-compatible nanomaterial that provide sufficient sensitivity for MRI t2-weighted imaging (Ling and Hyeon, 2013; Zeng et al., 2017). Accordingly, SPION particles have found clinical usage (Bergamino et al., 2014). Specifically, the covalent decoration of superparamagnetic iron oxide with interleukin-6 receptor-targeting peptide allows the particle to pass through the blood brain barrier, making it useful for the identification of low-grade glioma (Tan et al., 2019).

**Figure 2:**
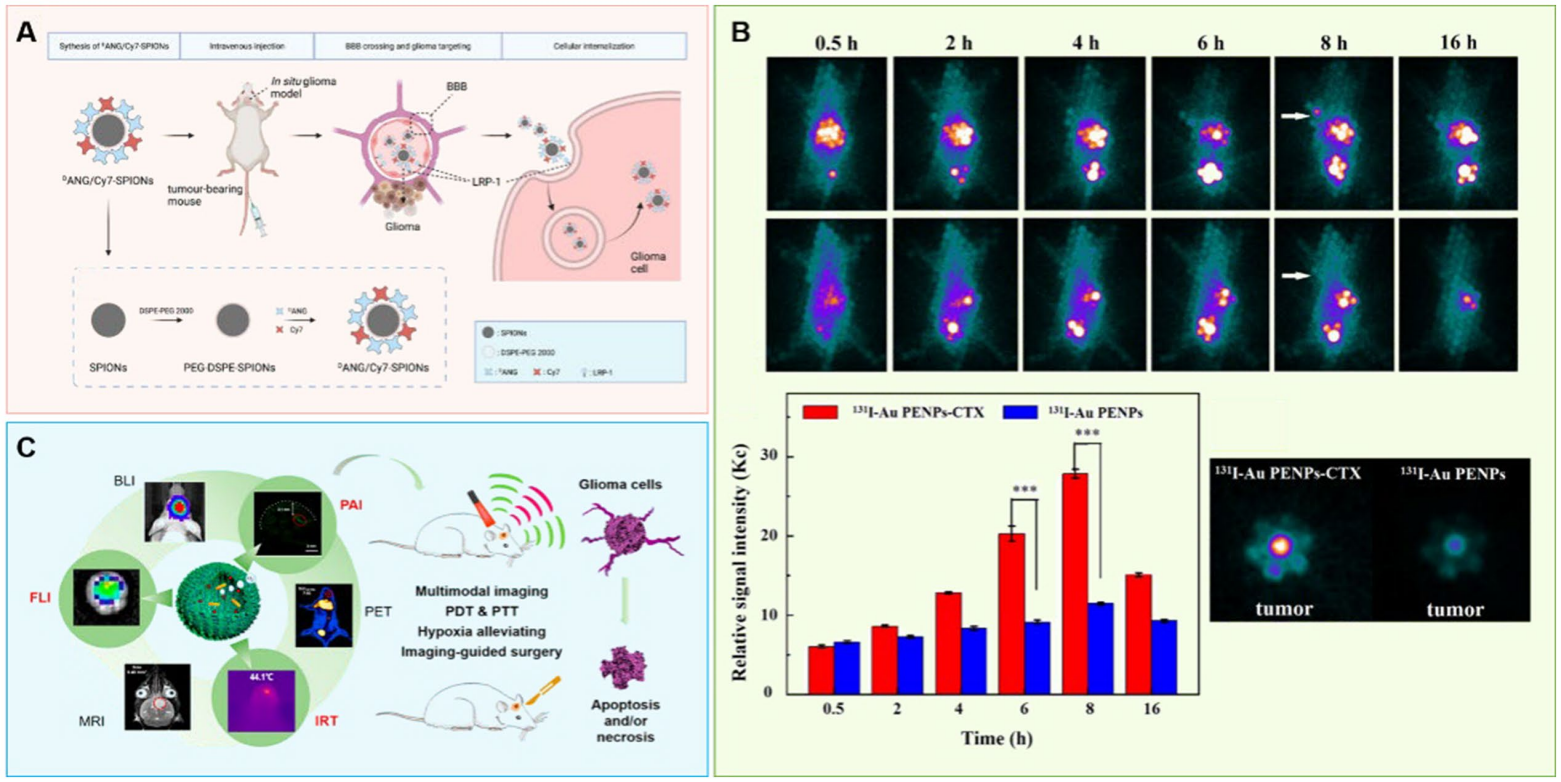
Using nanotechnology to achieve accurate diagnosis of brain glioma. **(A)** A nanoprobe SPIONs modified with Cy7 and ^D^ANG for targeted magnetic resonance/fluorescence imaging of glioblastoma. **(B)** Chlorotoxin peptide-functionalized polyethylenimine-entrapped gold nanoparticles for glioma SPECT/CT imaging and radionuclide therapy. **(C)** Albumin-based nanotheranostic probe for multimodal Imaging and phototherapy for glioma.

Any simple imaging method will have limitations, so it is necessary to find and develop multi-mode imaging with nanoparticles that can combine multiple imaging methods to diagnose GBM more accurately. For example, MnO NPs (nanoparticles) with excitation dependent fluorescence have been synthesized by thermal decomposition of manganese-based compounds to be used as nanoprobes for MRI imaging (Lai et al., 2018); this application combines MRI and fluorescence imaging without further coupling or encapsulation of imaging agents, giving this biocompatible nanomaterial with bimodal imaging a great potential for development. For example, Xie R. et al. (2021) developed a nanoprobe for MRI/NIR (near-infrared) fluorescence dual-modality imaging with SPIONs modified with indocyanine (Cy7) molecules and peptides (ANG or DANG) (Figure 2A). This probe exhibited excellent tumor-homing properties and barrier penetration *in vitro*, and mediated precise aggregation of nanoprobes at glioma sites in both *in vivo* MRI and *ex vivo* NIR fluorescence imaging. This strategy promises to improve preoperative precision imaging of GBM and to provide precise intraoperative display of GBM for real-time imaging.

### 3.2. Computerized tomography nanoprobe

Electronic computed tomography (CT) is relatively inexpensive, yet it has a high resolution and wide application range. Related applications include single electron emission computed tomography (SPECT; Greta et al., 2022), positron emission CT (PET) and X-ray CT.SPECT and PET are both methods of imaging that involve delivering radionuclides into the brain and then performing computational analyses of the distribution of radioactivity (Polyak and Ross, 2017). Nanomaterials can be co-labeled with radioisotopes and targeted molecules to increase the amount of contrast agents reaching the GBM site. It has been reported that the injection of nanomaterials labeled with radioisotopes into the GBM site can both image and treat GBM (Silindir et al., 2012).

X-ray (CT) involves irradiation of the body with X-ray, and it images the differential resistance of different tissues to X-rays (Chien et al., 2012). Since there is little difference in X-ray resistance among soft tissues, contrast agents often need to be added. At present, the most commonly used CT contrast agent is iodine agent, which is easily excreted but which has a short imaging time and leads to some side effects (Liu et al., 2012). On the other hand, gold nanoparticles (Kim et al., 2004; Jackson et al., 2010; Orta et al., 2021) have been shown to provide clear and durable images (Au et al., 2013) with better stability, longer residence time *in vivo*, more obvious contrast effect, and a stronger imaging ability (Porter et al., 1998; Cormode et al., 2014). These particles are relatively easy to modify, as well. For example, Zhao et al. selected polyethyleneimine as the template and sequentially modified it with polyethylene glycol (PEG) and a glioma specific peptide (chlorotoxin, CTX), and then embedded this complex onto gold nanoparticles (Au NP) (Zhao et al., 2019). These particles were useful as a nano probe for *in vitro* and *in vivo* targeted SPECT/CT imaging and radionuclide therapy of glioma cells in subcutaneous tumor models (Figure 2B). This nanoprobe was also shown to penetrate the BBB and specifically target glioma cells in rat intracranial glioma model.

### 3.3. Photo thermotherapy nanoprobe

Photothermal therapy (PTT) uses photothermal converters accumulated in a tumor to absorb light energy and generate heat that ablates tumor cells. Photothermal nanoprobes can realize fluorescence, photoacoustic and infrared thermal therapy as well as thermal imaging with high detection depth, high imaging contrast and high spatial resolution. It can distinguish tumor tissue from normal tissue and achieve precise diagnosis of glioma (Shang et al., 2017). The combined therapy and imaging functions of photothermal nanoprobes makes them a promising nanoplatform.

Nanomaterials with strong absorption in the near-infrared region (Wei et al., 2016) (including Au NPs, pyrazino [2,3-g]quinoxaline-based NPs; Zhang L.-P et al., 2021), and melanin nanoparticles (Marcovici et al., 2022) can be used as photoacoustic imaging contrast agents to produce a sharp contrast with the absorption imaging of biological tissues in the infrared light region. These nanomaterials tend to have high photothermal conversion rate, which can be useful in the photothermal treatment of tumors and can allow the integration of tumor diagnosis and treatment. The incorporation of biomimetics into nanomaterial biosensors is another step that has resulted in high detection speeds and accuracy, and it has great potential in tumor imaging (Telenga et al., 2019). Catalase biomimetics integrated with white egg white light thermotherapy enzyme nanoprobe can achieve multimodal imaging after passing through the BBB. During the imaging process, they can also release, decompose endogenous hydrogen peroxide into oxygen to alleviate the hypoxic state of glioma microenvironment, and improve cell apoptosis; these effects have been shown to extend survival times (Figure 2C; Yang et al., 2020).

The introduction of fluorescent nanomaterials as probes for tumor imaging (Huang et al., 2017; Wu et al., 2020) and the use of fluorescent nanomaterials as carriers of chemotherapy drugs represent another mechanism to achieve the integration of tumor diagnosis and treatment (Zhao et al., 2019). Notably, temperature control is a major limitation of standard applications of thermotherapy technology (Wei et al., 2016), but multimodal imaging combined with multiple imaging methods is a promising way to achieve precise diagnoses of GBM.

## 4. Precise treatments of GBM with nanotechnology

The treatment of brain tumors is challenging due to the difficulty of surgical resection, low drug efficacy and high recurrence rate (Murray et al., 2015). Current treatment strategies still rely on small or large molecule therapeutic drugs. The BBB serves as a potent defense mechanism to protect the brain from external and internal aggressors (Ballabh et al., 2004). Accordingly, its tight protein connection cause a high selectivity (Abbott et al., 2010; Saraiva et al., 2016) that allows only a very small number of hydrophilic small molecule drugs to cross into the BBB and reach a lesion; most macromolecular chemotherapeutics instead accumulate elsewhere in the body and damage healthy cells (Wohlfart et al., 2012). A hot topic of research in neurooncology is the development of targeted therapies to cross the BBB (Zhang et al., 2022) and deliver drugs to the tumor site. Advances in nanotechnology have piqued the interest of researchers who are developing targeted therapeutic carriers that can improve the efficiency of drug transport across the BBB and lead to innovative nanomedicine-based strategies (Zhang et al., 2022) Macromolecules can enter the central nervous system through the BBB through selective carriers and receptors (Nowak et al., 2019) in three main ways: (1) through vector mediated endocytosis; (2) through adsorption mediated endocytosis; (3) through receptor-mediated endocytosis. This review focuses on the study of nanomaterials developed to take advantage of receptor-mediated endocytosis for targeting GBM.

**Figure 3:**
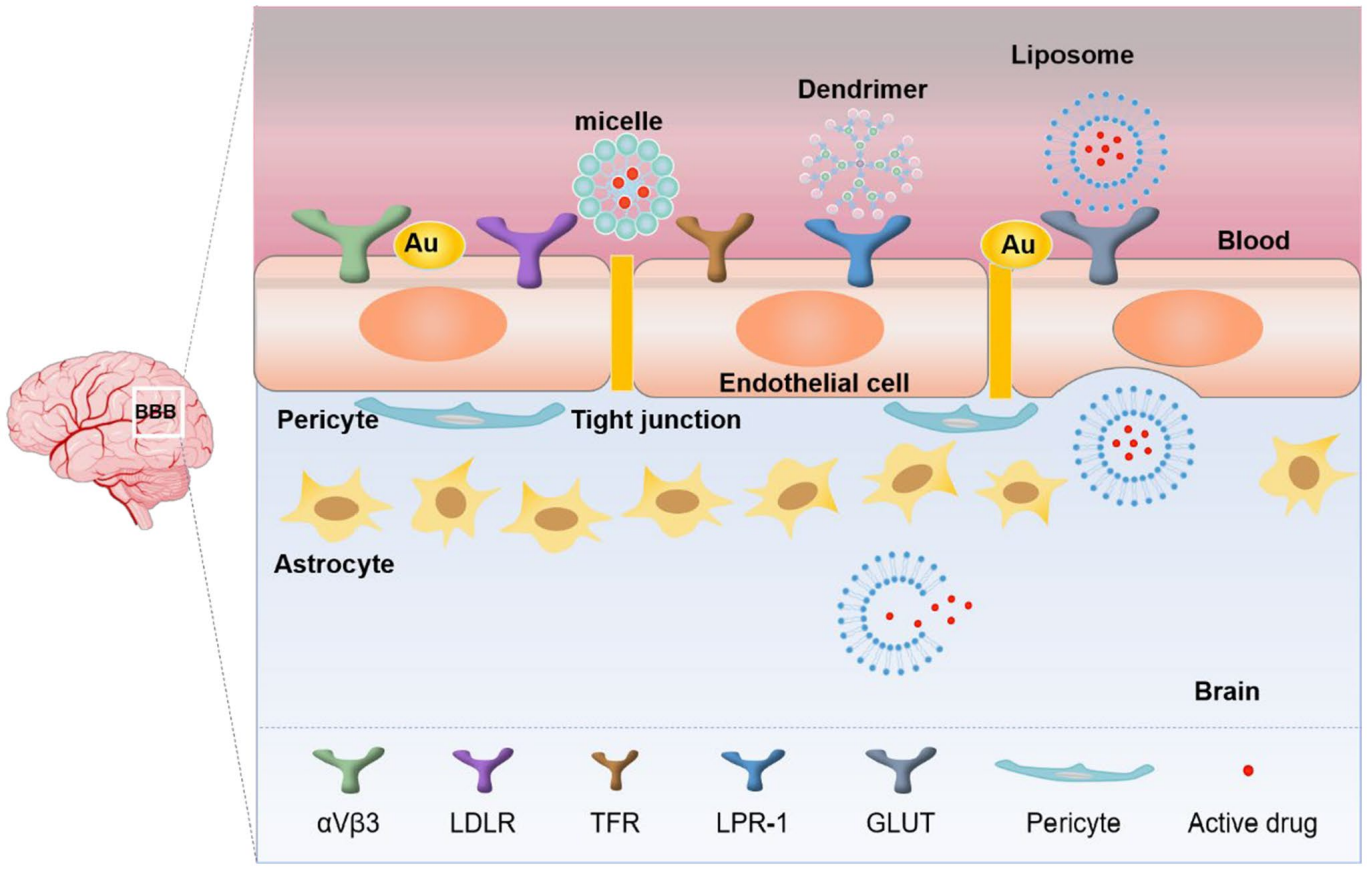
Receptor-mediated crossing of the BBB by Nanoparticles.

There are many receptors on the surface of the BBB, these receptors tend to be highly specific for cognate target molecules. The application of nanoparticles as drug carriers (Tosi et al., 2019), combined with compounds targeting specific receptors on the BBB, can provide targeted transport of hydrophobic macromolecular drugs to the site of the GBM. (Figure 3). The key receptors associated with the BBB are αVβ3, low-density lipoprotein receptor (LDLR), transferrin receptor (TFR), lipoprotein receptor related protein 1 (LPR-1), and glucose transporters (GLUT). Appropriate nanomaterials need to be selected as carriers of targeting molecules to combine with these receptors so that drugs can pass through the BBB to reach specific lesion areas and achieve targeted therapy (Furtado et al., 2018; Xie et al., 2019). Importantly, because tumor cells are heterogeneous, the correct nanomaterials should be selected according to the different tumor types for individualized therapy (Ramón et al., 2020; Carlberg et al., 2021).

The strategies used by researchers to move nanoparticles across the BBB are proliferating. Nanoparticles can carry drugs through the BBB and enrich in the area of GBM, improving the bioavailability and reducing toxic side effects. Macrophages entering the central nervous system is closely related to the destruction of the BBB (Ladewig et al., 2009). Using nanomaterials as drug carriers can reduce the number of drug molecules encapsulated by macrophages, so that drugs can be concentrated in the lesion area and targeted treatment can be achieved.

Nanomaterials include organic nanomaterials (such as liposomes and polymer nanoparticles) and inorganic nanomaterials (such as metal nanoparticles, carbon nanotubes and quantum dots). Nanodrugs that have been explored in clinical research and even brought to include liposomes, polymer nanoparticles and other organic nanomaterials. The following section introduces the organic nanomaterials with the highest biocompatibility and biodegradability: liposomes, polymer nanoparticles, and extracellular vesicles.

### 4.1. Nanoliposomes

Liposomes are small spherical vesicles (Shaw et al., 2017) with single or multiple lipid bilayers made of natural or synthetic lipids. The phospholipid bilayer structure of liposomes is compatible with the lipid layer of the BBB (Agrawal et al., 2017), and liposomes tend to be both non-toxic and biodegradable carrier (Kateb et al., 2011; Wen et al., 2012). Liposomes, which are considered self-assembling colloidal nanocarriers, can be used as carriers of preoperative and postoperative chemotherapeutic drugs for GBM and allow targeted drug delivery to glioma tissue. For example, several anticancer chemotherapeutics, including daunorubicin and doxorubicin (Han et al., 2017), have been incorporated into liposomal vesicles, which have been shown to be transported into glioma tissue (Zhao et al., 2016).

Nanoparticles can form tight interactions with plasma proteins, which then generate a protein corona (PC) that mediate their interaction with cellular and biological barriers (Cox et al., 2018). Apolipoprotein E (ApoE), an endogenous protein, is involved in lipid transport between brain and plasma (Li et al., 2020). Drug delivery can be mediated by the interaction of ApoE with the LDL receptor (LDLr) and LDLr-related protein 1 receptor (LRP1r) on cancer cells. Based on this idea, a strategy for an ApoE-rich PC-mediated brain-targeted drug delivery system for glioma therapy was developed (Zhang Z. A. et al., 2021). Aβ-CN peptide was used as a ligand for the formation of ApoE-rich PCs with brain-targeting properties. ApoE PC thus plays an important role in targeting BBB and delivering drugs to glioma cells.

Lactoferrin (LF) is an iron binding protein (Gruden and Poklar Ulrih, 2021) that can bind to transferrin receptors, such as TFR and CD71, that are overexpressed in tumor cells. Therefore, drug carriers modified with LF are able to cross the BBB *via* receptor-mediated endocytosis. LF has been added *via* passive drug loading into a lipid bilayer of magnolol, which is extracted from magnolia root and has a strong antiangiogenic effect. LF has been added *via* passive drug loading into a lipid bilayer of magnolol, which is extracted from magnolia root and has a strong antiangiogenic effect. These LF-modified nanocarriers then were induced to entrap daunorubicin *via* an active drug loading method. These nanocarriers were shown to inhibit angiogenesis mimicking channels (VM) and block the source of nutrients that fuel tumor cell invasion, and thus blocked tumor cell invasion (Liu et al., 2017).

Another recent study improved on this single-targeting carrier by the introduction of dual-targeting liposomes to inhibit angiogenesis mimicking channels (VM). This improved carrier used 4-aminophenyl β-D-glucopyranoside (Glu), a glucose derivative that binds to overexpressed glucose transporters (GLUTs) on the BBB (Chen J. et al., 2017; Li et al., 2019) Another glucose derivative, 4-aminophenyl β-D-glucopyranoside-D-α-tocopherol polyethylene glycol 1,000 succinate (glu-tpgs1000), was conjugated to the nanoparticle as a receptor molecule to facilitate transport across the BBB. These molecules were assembled into nanoscale liposomes that carried daunorubicin and rofecoxib, and the resulting liposomes were shown to be transported through the BBB *via* receptor-mediated endocytosis and adsorption-mediated endocytosis to deliver daunorubicin and the VM channel inhibitor rofecoxib (Xie H.-J. et al., 2021).

Cancer stem cells (CSCs) promote drug resistance by preferentially upregulating DNA damage checkpoint proteins (Afify and Seno, 2019). Previous studies have shown that treatment with Paclitaxel (PTX) alone can significantly induce the expression of related molecular targets in resulting tumor cells. Recently, a combination therapy of PTX and siRNA has been studied (Zhang et al., 2020). Angiopep-2 (Ang2) can target LDLr-related protein (LRP) to actively penetrate into brain compartments. LRP is highly expressed on BBB surface, and Ang2 can enhance drug delivery to GBM cells through BBB. Similarly, A15 is a targeted RNA ligand that binds to CD133 (a marker of CSCs) to track CD133 cancer cells. Therefore, when iopep-2 and A15 are used as dual-targeting ligands to connect doubly modified cationic liposomes (DP CLPs), DP CLPs directly and selectively deliver surviving siRNA and PTX to CSCs. This technique has been shown to improve tumor drug resistance (Sun et al., 2018). Ang2 was also added to the surface of solid lipid nanoparticles used for doxorubicin (DTX) delivery, and this package showed better targeting effects for GBM than did free doxorubicina (Kadari et al., 2018).

Liposome radiation therapy (Ravi et al., 2019) can magnify the effect of radiation therapy so that the corresponding therapeutic effect can be achieved with lower dose of radiation. Liposomes can also limit the effect of the radiation to the interior of the tumor, protecting the surrounding normal tissue cells from damage. In a phase I trial of recurrent GBM, it was shown that rhenium-186 nanoliposomes (186RNL) could selectively deliver β-emitting radiation (Floyd et al., 2015) to tumors. The 186RNL was administered by convection enhanced delivery (CED), and repeated imaging was performed by SPECT/CT. This method was well tolerated; no dose limiting toxicity has been found, and clinical activity has been observed (Brenner et al., 2021; Floyd et al., 2021). CED is compatible with the delivery of liposome carriers, and it can replace intravenous injection (IV) to deliver drugs directly to the central nervous system (Louis et al., 2018) and may be particularly suitable to the treatment of GBM (Clark et al., 2017). Real-time CED of liposome-encapsulated irinotecan (ONIVYDE) has been used to drive the therapeutic drugs to the target by relying on the positive pressure gradient of continuous infusion. By including a co-flow contrast agent in the liposome, MRI can be used for real-time imaging monitoring (Kumar et al., 2019). The CED of nanoliposomes containing topoisomerase I inhibitor CPT-11(Mendiburu-Eliçabe and Gil-Ranedo, 2015) have been shown to reach the GBM to achieve precise treatment. However, the application of CED requires advanced technology, and large studies are needed to overcome some limitations of CED and to explore some unknowns.

### 4.2. Polymer nanoparticles

Polymer nanoparticles are promising materials for drug delivery. They can be classified according to their shapes, including polymer micelles, dendritic macromolecules, and polymer conjugates. Polymeric nanoparticles are stable and have good biocompatibility. They also have a high drug loading capacity and low production costs and are readily scalable and modifiable (Mahmoud et al., 2020). Polymer nanoparticles can be designed to encapsulate a drug and to be targeted to the tumor site (Kaffashi et al., 2017) to improve the bioavailability while protecting the drug from being degraded by blood protein or enzymes of the BBB.

Polymer micelles are vesicles (Clay et al., 2017; Cai et al., 2022) of nanometer diameter that are formed by self-assembly of amphiphilic polymers with hydrophilic and hydrophobic blocks. Various polymers can be induced to form micelles (Mohan et al., 2018), which infiltrate into the tumor microenvironment *via* junctions of damaged endothelial cells and are then internalized. This is now considered as a potential force for the precision treatment of GBM (Ghosh and Biswas, 2021).

When selecting amphoteric blocks, polyethylene glycol (PEG) is typically chosen as a hydrophilic block to combine with different hydrophobic blocks to form conjugated polymers. Combination therapies of targeting GBM with peptide-modified polymer micelles are better able to access the target GBM by moving through the BBB (Ran et al., 2017). The internalized RGD peptide (iRGD peptide) (Yin et al., 2017) contains an RGD motif and a protease recognition site. The RGD motif can associate with combinations of receptor integrins (αvβ3 or αvβ5) that are overexpressed on tumor vascular endothelial cells.(Duong and Coleman, 2002) Tumor-derived proteases then cleave the peptide and exposes a previously hidden C-end rule motif, which binds to the highly expressed neurodermin-1 (NRP-1; Zhuo et al., 2019) on the surface of GBM cells. Therefore, an iRGD-functionalized polymer prodrug micelle system can achieve high tumor penetration (Kang et al., 2020). Lu et al. (2020) developed a disulfide-linked pre-drug polymer consisting of camptothecin (CPT) and PEG, and further modified the iRGD peptide. PEG and the chemotherapy drug CPT were used to form a conjugated precursor polymer, and then combined with photosensitizers to self-assemble into a 100 nm diameter nanopolymer micelle. The RGD peptide was coupled and internalized on the surface of the micelle, and this particle inhibited tumor growth and reduced side effects. Other *in vitro* and *in vivo* studies have shown that targeted prodrug systems can not only effectively cross various barriers to reach GBM sites, but can also significantly enhance the antitumor effect by laser irradiation. Nanomicelles formed by PEG and PTX polymer modified with synthetic TfR-T12 peptide can be rapidly absorbed by tumor cells and inhibit GBM (Sun et al., 2020).

Dendrimers are highly branched and structurally precise molecules synthesized by repeated growth reactions (Dias et al., 2020). Dendrimers are regularly hyperbranched macromolecule (Sheveleva et al., 2021) that can covalently bind a large number of drugs to nanoparticles. Dendrimer nanoplatforms designed to attach to target ligands can modulate drug release rate, increase drug permeability, and direct specific drugs to their targets (Chauhan, 2018; Zou and He, 2021). The most common dendrimer is polyamide amine (PAMAM), which has high solubility and low toxicity (Filipczak et al., 2021). PAMAM dendrimer-based carriers are considered to be promising drug-targeting carriers for cancer therapy due to their rich peripheral amino groups and internal cavity structure, which can be modified by various ligands and encapsulate many chemotherapeutic drugs (Bertrand et al., 2011; He et al., 2011). Similarly, the EP-1 peptide has high affinity and specificity for epidermal growth factor receptor (EGFR), and it can be used to enhance tumor targeting efficiency. Accordingly, PAMAM dendrimer carrier have been modified with both Ang2 and EP-1 peptides. This dual-targeted dendrimer with enhanced BBB permeability and higher targeting efficiency significantly enhances the anti-glioma effect of the drug and prolongs the survival period of mice with hormonal GBM (Dwivedi et al., 2016; Liu et al., 2019).

### 4.3. Extracellular vesicles

Extracellular vesicles (EV) are a group of heterogeneous cell-derived membrane structures that provide a mechanism for intercellular communication and allow cells to exchange proteins, lipids, RNA, and other genetic material (van Niel et al., 2018). The two known types of EV are exosomes and microvesicles. Exosomes have diameters of 40 to 100 nm and can be produced by multiple cell types, such as dendritic cells (DC), fibroblasts, neuronal cells, stem cells and cancer cells (Wu et al., 2021). In the brain, exosomes have been shown to transfer nucleic acids, proteins and lipids between neurons and glial cells, and they are also key mechanisms of transfer of information between these cells (Murgoci et al., 2018).

Tian et al. (2022) used EVs loaded with small interfering RNA (siRNA) targeting programmed cell death ligand-1 (PD-L1) as an immune checkpoint blockade. A brain-tumor-targeting cyclic peptide (RGDyK) was also selected to modify the EV surface and to enhance accumulation using radiation bursts, resulting in enhanced delivery of EV to GBM. This EV-based strategy was showned to significantly improve the targeting efficiency of GBM. The loaded siRNA reversed radiation-stimulated PD-L1 expression on tumor cells and recruited tumor-associated bone marrow cells with synergistic effects. The combination therapy significantly increased the activity of CD8 cytotoxic T cells, arrested tumor growth and prolonged the survival of the animals.

Wu et al. (2022) developed a novel exosome-based delivery system targeting GBM for the enhancement of sonodynamic therapy (SDT). Catalase (CAT) was encapsulated into silica nanoparticles (CAT@SiO_2_) which were then loaded with indocyanine green (ICG) as a sonosensitizer. Inspired by the ability of macrophages to cross the BBB, CSI was further encapsulated with AS1411 aptamer-modified macrophage exosomes to form CSI@Ex-A nanoparticles. High levels of reduced glutathione triggered the biodegradation of CSI@Ex-A, and the released CAT catalyzed the production of O_2_ from H_2_O_2_ to alleviate the intracellular hypoxia. The nanoparticles exhibited efficient BBB penetration and a good cancer cell-targeting ability. In addition, depletion of glutathione and the supply of O2 improved the efficiency of SDT, and the nanoformulation showed low toxicity to normal cells and tissues. Thus, this nanoplatform may offer great promise for clinical applications (Table 1).

**Table 1:** Nanocarrier-based strategies for GBM treatment *via* receptor-mediated therapy.

Nanocarrier	Receptor	Ligand	Drug	Reference
Liposomes	LDLR	ApoE	Paclitaxel	Zhang et al. (2021)
	TFR	LF	Daunorubicin	Liu et al. (2017)
	GLUT	Glu-tpgs1000	Daunorubicin, Rofecoxib	Xie et al. (2021)
	LPR1	Angiopep-2	Paclitaxel, Doxorubicin	Kadari et al. (2018); Sun et al. (2018); Zhang et al. (2020)
Polymer nanoparticles	αvβ3	RGD	Calprotectin	Lu et al. (2020)
	TFR	TfR-T12 peptide	Paclitaxel	Sun et al. (2020)
	LPR1	Angiopep-2	Doxorubicin	Liu et al. (2019)
Extracellular vesicles	αvβ3	RGD	Programmed cell death ligand-1 siRNA	Tian et al. (2022)

## 5. Challenges in the treatment of GBM with nanotechnology

Nanotechnology has provided unique contributions to the field of diagnosis and treatment of GBM. It is worth noting that safety is very important in the application and development of nanomaterials, and it is a prerequisite for the application of nanomaterials for the treatment and diagnosis of GBM. Before nanomaterials are applied to the human body, it is necessary to ensure that the nanoparticles themselves are non-toxic. The extent of toxicity of a nanoparticle is associated with size, shape, motion, properties, stability, media, and storage timeT (Sufian et al., 2017). Nanoparticles exhibit a large surface-to-volume ratio due to their ultra-small size. This property means that nanoparticles release cargo rapidly and thus can lead to toxicity (Donaldson et al., 1998). Similarly, the shape and charge on the nanoparticles can facilitate translocation through the cell membrane (Hagens et al., 2007). Organic nanomaterials, especially liposomes, have been studied extensively and in some cases approved for clinical use. However, studies on the *in vivo* toxicity of inorganic nanomaterials have generally only lasted for several days up to several weeks; thus, there is a lack of assessment of long-term consequences of treatment with these materials (Allen, 2019). Another issue involves the individual nature of cancer: the tumors of different patients at different stages have different properties. It is thus necessary to design flexible nanoparticles that can be altered to be suitable for a variety of situations. The production of individualized nanocarriers is quite complicated and the production cost is high, which makes it difficult to achieve large-scale production. The penetration depth of nanoparticles also cannot be guaranteed, which has made the conversion to clinical applicability difficult. Laws and regulations related to nanotechnology management are not perfect, and ethical issues related to nanotechnology need to be considered.

## 6. Conclusion

The difficulties to be overcome in the treatment of GBM have been: (1) the limited penetration of drugs through the BBB to the brain parenchyma; (2) High postoperative recurrence rate due to chemical resistance caused by stem cell like characteristics; (3) The toxic and side effects of low selectivity of drugs on normal nerve cells. Although there are currently various strategies and schemes for enriching nanoparticles through the BBB into lesions, approved clinical drugs for the treatment of solid tumors mainly rely on passive targeted therapy, while dense brain matrix in GBM can hinder diffusion, so passive targeted therapy is likely to be ineffective. Direct injection of drugs into the brain using stereotactic therapy or passive targeting therapy cannot effectively penetrate GBM infiltrating tumor cells. Therefore, future treatment of GBM should focus on active targeted therapy.

Overall, nanotechnology has opened up a new path for the diagnosis and treatment of GBM. The field is still new, and this road can be broadened. However, the application of nanotechnology remains limited throughout the treatment process. The toxicity, stability, biocompatibility, biodegradability of nanomaterials and the ethical issues of the application of nanotechnology to the human body still need to be further studied and clarified.

## Author contributions

DW, JL, and NZ designed this study. DW, NZ, SQ, and HW drafted this manuscript. All authors contributed to the article and approved the submitted version.

## Funding

This study was supported by grants from National Key R&D Program of China (2022YFC2009906 and 2022YFC2009900), 1.3.5 Project of West China Hospital, Sichuan University (ZYJC21029), Post-Doctoral Research Project, West China Hospital, Sichuan University (20HXBH040), and Project funded by the China Post-Doctoral Science Foundation (2021M692293).

## Conflict of interest

The authors declare that the research was conducted in the absence of any commercial or financial relationships that could be construed as a potential conflict of interest.

## Publisher’s note

All claims expressed in this article are solely those of the authors and do not necessarily represent those of their affiliated organizations, or those of the publisher, the editors and the reviewers. Any product that may be evaluated in this article, or claim that may be made by its manufacturer, is not guaranteed or endorsed by the publisher.
